# Controllable Hierarchical Mechanical Metamaterials Guided by the Hinge Design

**DOI:** 10.3390/ma14040758

**Published:** 2021-02-05

**Authors:** Krzysztof K. Dudek, Ruben Gatt, Miroslaw R. Dudek, Joseph N. Grima

**Affiliations:** 1 Institute of Physics, University of Zielona Gora, ul. Szafrana 4a, 65-069 Zielona Gora, Poland; m.dudek@if.uz.zgora.pl; 2 Metamaterials Unit, Faculty of Science, University of Malta, Msida, MSD 2080, Malta; ruben.gatt@um.edu.mt (R.G.); joseph.grima@um.edu.mt (J.N.G.); 3 Centre for Molecular Medicine and Biobanking, University of Malta, Msida, MSD 2080, Malta; 4 Department of Chemistry, Faculty of Science, University of Malta, Msida, MSD 2080, Malta

**Keywords:** hierarchical, auxetic, mechanical metamaterials, Poisson’s ratio

## Abstract

In this work, we use computer simulations (Molecular Dynamics) to analyse the behaviour of a specific auxetic hierarchical mechanical metamaterial composed of square-like elements. We show that, depending on the design of hinges connecting structural elements, the system can exhibit a controllable behaviour where different hierarchical levels can deform to the desired extent. We also show that the use of different hinges within the same structure can enhance the control over its deformation and mechanical properties, whose results can be applied to other mechanical metamaterials. In addition, we analyse the effect of the size of the system as well as the variation in the stiffness of its hinges on the range of the exhibited auxetic behaviour (negative Poisson’s ratio). Finally, it is discussed that the concept presented in this work can be used amongst others in the design of highly efficient protective devices capable of adjusting their response to a specific application.

## 1. Introduction

Mechanical metamaterials [1,2,3] are a class of rationally-designed systems that can exhibit counter-intuitive mechanical properties based on their design rather than material’s composition. Over the years, these systems have been thoroughly analysed from the point of view of their potential to exhibit unusual mechanical properties such as auxetic behaviour [4,5,6,7,8,9,10,11,12], negative compressibility [13,14,15,16,17,18,19,20] and negative stiffness [21,22,23,24,25,26]. This large and continuously growing interest of the research community in the mechanical metamaterials stems from the fact that their unusual mechanical properties can be utilised in numerous applications ranging from biomedical [27] to soundproofing [28,29] and protective devices [30,31,32,33].

Over the years, a large number of different classes of mechanical metamaterials have been proposed with all of them being capable of exhibiting unusual mechanical behaviour. It seems that some of the most studied examples of such structures include rotating rigid unit systems [34], re-entrant structures [35,36] and chiral systems [37,38,39,40,41]. However, it is important to emphasise the fact that all of these classes of mechanical structures as well as many other types of mechanical metamaterials known in the literature have one limitation. Namely, once they are manufactured, it is difficult to change their mechanical properties or a deformation pattern. In fact, in a majority of mechanical metamaterials, to achieve such effect, it would be necessary to destroy some elements of the structure whose process is clearly not reversible. In view of this, a majority of popular types of mechanical metamaterials cannot normally act as controllable devices capable of adjusting their response to a specific application. Fortunately, there is another class of these systems called hierarchical mechanical metamaterials [42,43,44,45,46,47,48,49,50,51,52], where, as reported in recent years, such effect is possible.

Hierarchical mechanical metamaterials are a class of systems where substructures composing the system have their independent geometry. This means that the structure can undergo a multi-level deformation process which in general can result in a variety of different types of mechanical behaviour. The possibility of controlling the behaviour of hierarchical metamaterials was first discussed by Gatt et al. [44] and Cho et al. [45] in which cases the multi-level rotating squares system was taken into account. This concept was later explained through a simple theoretical model by Dudek et al. [50] and further developed by other research groups with the emphasis on experimental studies [53,54,55]. However, studies related to hierarchical mechanical metamaterials are still in their infancy and, in order to achieve a full control over their behaviour, it is necessary to acquire a much deeper understanding of mechanisms governing their deformation process. Thus, an analysis of the dynamic behaviour of hierarchical mechanical metamaterials could lead to the design of structures that could fine-tune their mechanical response depending on a particular application. This in turn could be beneficial from the point of view of the industry and potential applications as it would help to increase the efficiency of numerous currently known materials.

In view of the above, in this work, we are going to study the dynamic behaviour of a particular hierarchical metamaterial in order to assess the possibility of achieving a control over its behaviour. To achieve it, we are going to analyze the possibility of controlling the behaviour of specific parts of the structure upon using different types of hinges depending on their specific location within the system. Finally, we are going to investigate the mechanism responsible for specific hierarchical levels of the system deforming at a different rate than the others which could prove to be very significant in the design of other hierarchical mechanical metamaterials acting for example as efficient protective devices.

## 2. Methods

### 2.1. Geometry

**Figure 1:**
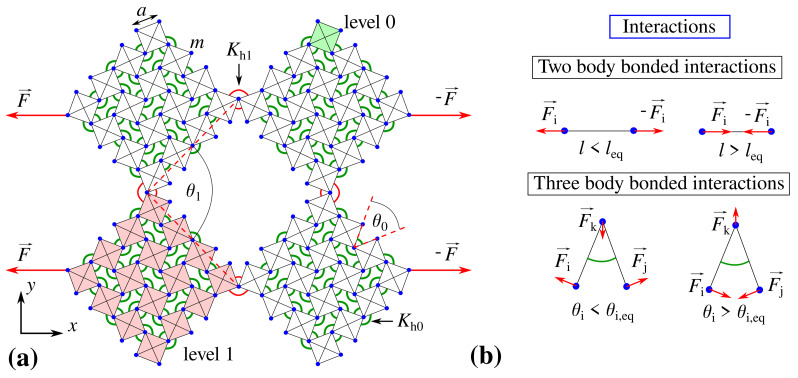
The panels show (**a**) an example of the analyzed system corresponding to $N_{x}\times N_{y}=4\times 4$ where red arrows indicate constant external forces applied to the leftmost and rightmost points within the system and (**b**) a graphical representation of interactions responsible for rigidity of square elements (two-body bonded interactions) and hinging between them (three-body bonded interactions).

In this work, a two-dimensional hierarchical mechanical metamaterial composed of square-like structural units is investigated from the point of view of its potential to exhibit a controllable mechanical behaviour (see Figure 1). The considered system has two levels of hierarchy where level 0 of the structure corresponds to individual squares having a linear dimension of *a* while level 1 represents larger building blocks consisting of $N_{x}\times N_{y}$ level 0 elements. Furthermore, all of the level 0 structural units have a discrete mass distribution where only points at the corners of respective structural units have a non-zero mass, namely mass *m* (see Figure 1a). Different pairs of these points are connected by means of stiff bond-like elements having their length governed by the harmonic potential associated with the stiffness constant *k* (see Section 2.3) so that square-like structural units can retain their shape throughout the entire deformation process. In addition, in order to reliably mimic the behaviour of elastic systems, it is assumed that the hinging motion exhibited by different pairs of level 0 elements corresponds to some non-zero resistance of these elements to the rotational motion which changes with the extent of the mechanical deformation. To achieve it, the hinging motion is assumed to be governed by the harmonic potential associated with the stiffness constant $K_{h,i}$ where the value of this parameter can change depending on the location of a particular hinge within the system. More specifically, in the considered structure, it is possible to distinguish two types of hinges, i.e., hinges where the stiffness constant assumes the value of $K_{h0}$ or $K_{h1}$ (see Figure 1a). In this work, it is assumed that hinges connecting level 0 elements being a part of the same level 1 block always correspond to the same stiffness constant $K_{h0}$. On the other hand, the four hinges connecting larger level 1 building blocks are associated with the $K_{h1}$ parameter (see Section 2.3). It is also important to note that the behaviour of all of the hinges is represented by appropriate three-body bonded interactions as graphically portrayed in Figure 1b. At this point, it is also worth mentioning that, even though in this work we only consider the harmonic potential to describe the hinging motion of structural elements, in general, one could take an arbitrary type of the potential into account. In addition, results could vary from those reported in this study if a different potential is used.

Based on Figure 1a, it is possible to note that the considered system may assume a plethora of different configurations. However, due to a large rigidity of square units, one can note that there are only two geometric parameters that determine a particular configuration of the structure, i.e., angles $\theta_{0}$ and $\theta_{1}$. Thus, the deformation process induced by external forces may result only in the change in these two parameters which in turn leads to the deformation of the level 0 and level 1 of the system. More specifically, the opening of the level 0 of the structure results in the increase in the value of $\theta_{0}$ and the increase in the linear dimensions of level 1 blocks. On the other hand, the opening of the level 1 of the structure results in the increase in the value of $\theta_{1}$ and the rotation of large building blocks shown graphically in Figure 1a by means of the red colour. Nevertheless, it is important to note that, due to its geometry, the system cannot assume the configuration corresponding to an arbitrary combination of angles $\theta_{0}$ and $\theta_{1}$. In fact, there is a very limited range of these angles that can be assumed by the system without the structural units overlapping. Such conditions [50] can be expressed in a following manner: $\theta_{1}>\theta_{0}$, $\pi -\theta_{1}-\theta_{0}>0$ and $\theta_{0},\theta_{1}\in \left[0,\pi \right]$. Thus, in order to have a possibility of observing a significant evolution of both levels of the structure, in this work, the initial configuration of the structure subjected to the mechanical deformation is assumed to always correspond to the angles $\theta_{0}=10^{\circ }$ and $\theta_{1}=40^{\circ }$.

### 2.2. Deformation Process

In order to assess the mechanical properties of the considered system, it is subjected to the mechanical deformation induced by constant external forces. These forces have a magnitude *F* and are applied to the two leftmost and two rightmost vertices of the structure as shown in Figure 1a. It is important to note that, as the system deforms, the positions of the outmost vertices of the structure change. In this work, we assume that the points where external forces are applied shift together with the outmost vertices of the structure so that the system is not constrained from the motion in any direction during its deformation process. However, it is interesting to note that, if the external forces were not allowed to shift together with the system, it could induce the out-of-plane buckling of the structure as discussed in other studies [53]. Finally, it is important to emphasize the fact that, in this work, the evolution of the structure which is initially at rest is analyzed only up to the point where neighbouring structural units collide or when the external forces are no longer large enough to overcome the elastic restoring forces corresponding to the system. At this point, it is worth noting that, in this work, we assume that the external forces applied to the system can only result in the deformation of the structure (i.e., primarily the rotation of square units) and cannot damage it or its components. In practice, it is envisaged that, should the applied loads be excessive in magnitude, this assumption may not hold.

### 2.3. Simulations and Parameters

In this work, in order to analyze the evolution of the considered system, we conduct Molecular Dynamics (MD) simulations utilising the fourth order Runge–Kutta method [56] for a constant time step $\Delta t=10^{-6}$ s. In this case, the points constituting the system in addition to the external forces also experience forces originating from the bonded interactions present within the system. More specifically, two-bonded interactions are assumed to be governed by means of the harmonic potential $V_{2}=\frac{1}{2}k{\left(l-l_{eq}\right)}^{2}$, where *k* is the stiffness constant and, for all of the simulations, it assumes the value of $2\times 10^{6}$ Nm${}^{-1}$. In addition, *l* is the current length of a given bond-like element and $l_{eq}$ is its equilibrium value. In our case, we assume that $l_{eq}=a=2$ cm. Furthermore, the three-body bonded interactions responsible for hinging between structural units are also assumed to be governed by means of the harmonic potential defined as $V_{3}=\frac{1}{2}K_{h}{\left(\theta_{i}-\theta_{i,eq}\right)}^{2}$, where $K_{h}$ is the stiffness coefficient and, depending on the particular set of results analysed in this work, it assumes a value from the interval between $K_{hS}=1.23\times 10^{-4}$ J deg${}^{-2}$ and $K_{hL}=0.314$ J deg${}^{-2}$. The remaining parameters used in this work were set to be the following: *m* = 40 g, *F* = 10 N. At this point, it is worth noting that the parameters were selected in a way so that the deformation time would match the time scale reported in the literature for experimental studies incorporating similar structures [57].

In this work, in order to assess mechanical properties of the considered system, we measure its Poisson’s ratio. To do this, we use the engineering definition of this quantity with its definition for loading in the *x*-direction being shown below:

(1) $$\nu_{xy}=-\frac{\varepsilon_{y}}{\varepsilon_{x}}=-\left(\frac{l_{y}\left(t\right)-l_{y}\left(t=0\right)}{l_{y}\left(t=0\right)}\right)/\left(\frac{l_{x}\left(t\right)-l_{x}\left(t=0\right)}{l_{x}\left(t=0\right)}\right)$$

In this case, $l_{x}\left(t\right)$ and $l_{y}\left(t\right)$ are the current dimensions of the structure in the *x*- and *y*-dimensions, respectively. On the other hand, $l_{x}\left(t=0\right)$ and $l_{y}\left(t=0\right)$ are initial dimensions of the system. At this point, it is also worth noting that all of the results were generated through the code written by the authors in the Python programming language.

At this point, it is worth mentioning that, in general, MD-type simulations have some limitations. First and foremost, they correspond to a non-zero numerical error which may affect generated results. Hence, in order to solve differential equations of motion describing the evolution of a given system, it is essential to select the appropriate numerical algorithm and the size of the corresponding time step in order to ensure that the size of the numerical error is insignificant. In view of this, in our work, in addition to a small time step referenced above, we incorporated a very stable fourth-order Runge–Kutta algorithm that allows us to ensure that the obtained results are reliable and that the corresponding numerical errors are negligible. Another limitation of this class of simulations corresponds to the use of a discrete mass distribution. However, through the use of appropriately defined bonds connecting point-like elements having a non-zero mass, it is still possible to represent the behaviour of different structural elements including rigid bodies. On the other hand, it is also important to emphasise the fact that MD simulations offer numerous advantages in comparison to other approaches. First of all, they allow for analysing the dynamic evolution of the structure even in the case of big strains. They also take into account the inertia of the system during such dynamics processes, whose effect is not taken into account in the case of many different simulations methods. Finally, the computation time corresponding to MD simulations is often significantly lower than is the case for other methods.

## 3. Results and Discussion

**Figure 2:**
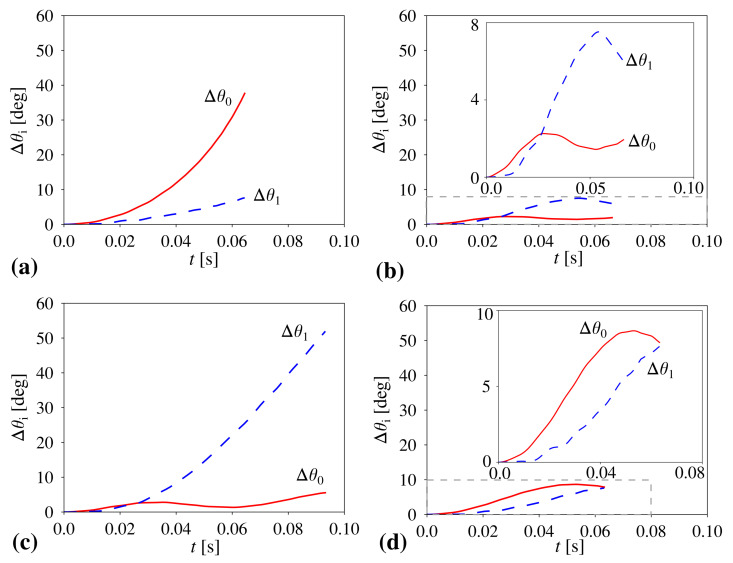
The panels show the behaviour of the system when hinges associated with level 0 and level 1 correspond to different values of a stiffness constant, namely: (**a**) $K_{h0}=K_{h1}=K_{hS}$ (all hinges are soft, $K_{hS}=1.23\times 10^{-4}$ J deg${}^{-2}$), (**b**) $K_{h0}=K_{h1}=K_{hL}$ (all hinges are stiff, $K_{hL}=0.314$ J deg${}^{-2}$), (**c**) $K_{h0}=K_{hL}$ and $K_{h1}=K_{hS}$ (level 0 hinges are stiff and level 1 hinges are soft), and (**d**) $Kh_{0}=K_{hS}$ and $K_{h1}=K_{hL}$ (level 0 hinges are soft and level 1 hinges are stiff). All of the results presented in this figure correspond to the system having level 1 structural blocks composed of $N_{x}\times N_{y}=4\times 4$ square units. In addition, $\Delta \theta_{i}=\theta_{i}\left(t\right)-\theta_{i}\left(t=0\right)$, where i=0 or i=1.

The main objective of this work is to show the possibility of achieving a control over the behaviour and mechanical properties of the considered hierarchical metamaterial. To do this, we analyse the effect of the variation in the magnitude of the stiffness coefficient corresponding to hinges connecting different structural units on the overall behaviour of the system and the evolution of respective hierarchical levels of the structure. To this aim, we first analyse the behaviour of the system where all of the hinges within the structure correspond to the same stiffness constant, i.e., $K_{h0}=K_{h1}$ (see Figure 2a,b).

**Figure 3:**
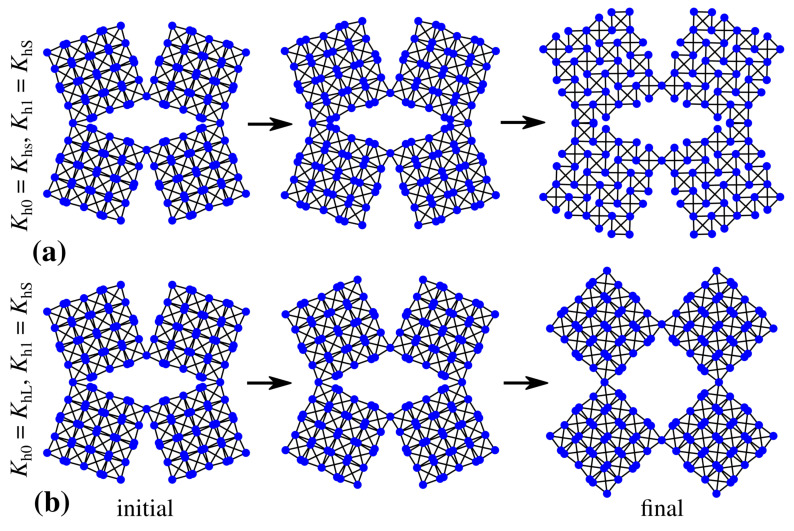
The panels show the evolution of the hierarchical system where: (**a**) $K_{h0}=K_{h1}=K_{hS}$ (all hinges are soft) and (**b**) $K_{h0}=K_{hL}$ and $K_{h1}=K_{hS}$ (level 0 hinges are stiff and level 1 hinges are soft).

Based on Figure 2a, one can note that, in the case of the system where all of the hinges are relatively soft and correspond to the same stiffness constant $K_{h0}=K_{h1}=K_{hS}$ (see Section 2.3), level 0 of the structure opens to a significantly larger extent than level 1. This is manifested by a much larger increase in the value of $\theta_{0}$ than $\theta_{1}$. Such behaviour of the system originates from the fact that, in the case of a negligible or very small magnitude of the stiffness coefficient, the governing factor determining the rate of expansion of respective structural levels becomes the torque corresponding to external forces applied to specific vertices within the structure. As discussed in [50], due to the particular geometry of the two-level hierarchical square system, the torque acting on level 0 elements is larger than in the case of the level 1 blocks, which results in the angle $\theta_{0}$ opening at a faster rate than $\theta_{1}$. This, in turn, means that, during the deformation process, the rotation of level 1 building blocks is relatively small in comparison to the extension of linear dimensions of level 1 building blocks composed of level 0 squares (see Figure 3a).

According to Figure 2b, it is possible to see that the behaviour of the system presented in Figure 2a becomes completely reversed in the case when all of the hinges are stiff and correspond to the same stiffness coefficient $K_{h0}=K_{h1}=K_{hL}$ (see Section 2.3). More specifically, in this situation, angle $\theta_{1}$ increases to a much larger extent than $\theta_{0}$ at the end of the deformation process. This stems from the fact that, in order to open level 0 of the system, it is necessary to rotate a much larger number of hinges as opposed to level 1 of the structure, which is associated solely with four hinges. Thus, the resistance of hinges to the rotational motion is much larger for level 0 of the structure than is the case for level 1 of the system, which results in the situation where we primarily observe the rotation of level 1 building blocks while their linear dimensions remain almost unchanged. At this point, it is also important to emphasize the fact that the use of stiff hinges results in a smaller extent of the deformation of the entire system than is the case for the structure constituted solely by light hinges. This stems from the fact that, in both of these cases, the same external forces were used to deform the system. Thus, a much larger resultant stiffness of the system corresponding to the results shown in Figure 2b makes it more difficult for external forces to deform the structure.

In addition to structures having all of their elements connected by hinges corresponding to the same stiffness coefficient, it is also very interesting to consider a hybrid system where level 0 and level 1 are composed of hinges having different stiffness. As shown in Figure 2c, one such example can be the structure with soft hinges ($K_{h1}=K_{hS}$) connecting level 1 building blocks and stiff hinges ($K_{h0}=K_{hL}$) connecting level 0 squares. As a result, during the deformation process, the rotation of level 0 squares is negligible while level 1 building blocks rotate to a very significant extent corresponding to the change in $\theta_{1}$ being approximately equal to 52${}^{\circ }$ (see Figure 3b). On the other hand, as shown in Figure 2d, in the case of the hybrid system where level 0 elements are connected by soft hinges ($K_{h0}=K_{hS}$) and level 1 building blocks are connected by means of stiff hinges ($K_{h1}=K_{hL}$), very different behaviour can be observed. Namely, both level 0 and level 1 of the structure deform to a small extent. This stems from the particular design of the hinges where level 0 squares cannot rotate without all of the squares forming level 1 building blocks being able to rotate. This, in turn, is very important as based on Figure 1a, one can note that, in each level 1 building block, there is always one level 0 square that is connected to one of its neighbours by means of the hinge corresponding to the $K_{h1}$ stiffness constant. Thus, even though the resistance between level 0 squares is relatively small, they cannot significantly deform due to the presence of the four stiff hinges.

**Figure 4:**
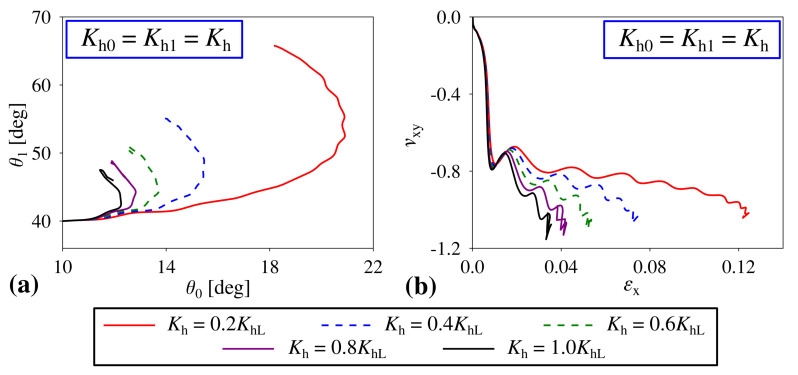
The effect that the variation in the stiffness coefficient related to the hinges has on the behaviour of the structure. (**a**) the dependence of $\theta_{1}$ vs. $\theta_{0}$ during the deformation process and (**b**) the variation in the Poisson’s ratio for loading in the *x*-direction plotted against strain.

Up to this point, we have only analysed structures where hinges were associated either with a large or small stiffness coefficient, i.e., $K_{hL}$ and $K_{hS}$ respectively. On the other hand, it is also very interesting to investigate the behaviour of the system where the stiffness constant corresponding to hinges would assume an arbitrary value from within this range. As shown in Figure 4, the variation in the value of the stiffness coefficient may significantly affect the deformation pattern exhibited by the structure as well as its mechanical properties. More specifically, based on Figure 4a, it is possible to note that structures where $K_{h}$ ($K_{h}=K_{h0}=K_{h1}$) assumes relatively small values have both of their hierarchical levels opening to a larger extent than for structures with large values of $K_{h}$. This is reasonable as the increase in the value of $K_{h}$ results in the larger stiffness of the entire structure. Furthermore, based on Figure 4b, one can note that the variation in the value of $K_{h}$ can be also used in order to control the value of the Poisson’s ratio exhibited by the structure. More specifically, the larger the value of $K_{h}$, the more auxetic the system. This stems from the fact that, for large values of this coefficient, level 1 blocks retain approximately a square-like shape throughout the deformation process. On the other hand, structures with softer hinges have their level 1 blocks deforming to a larger extent which results in them resembling a rectangle. This in turn leads to the increase in the value of the Poisson’s ratio in comparison to structures composed of square-like units.

**Figure 5:**
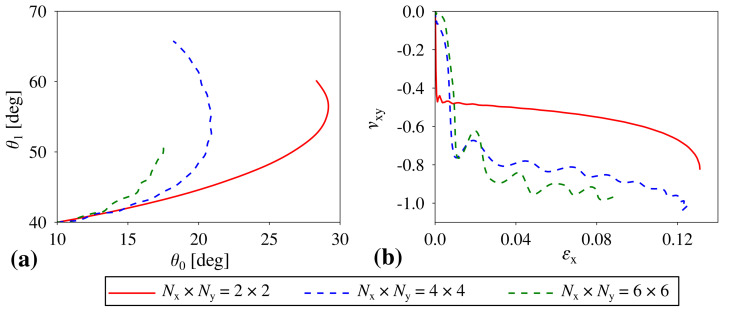
The effect of the variation in the number of squares constituting level 1 building blocks on the behaviour of the structure. These results were generated for structures where all off the hinges corresponded to the same stiffness coefficient equal to 0.2 $K_{hL}$. (**a**) the dependence of $\theta_{1}$ vs. $\theta_{0}$ during the deformation process and (**b**) the variation in the Poisson’s ratio for loading in the *x*-direction plotted against strain.

At this point, it is worth noting that all of the results presented in this study were generated for structures where level 1 building blocks were composed of 4×4 square elements, i.e., $N_{x}\times N_{y}=4\times 4$. Thus, it would be also very interesting to analyze the behaviour of the structure composed of a different number of units. As shown in Figure 5a, the external forces applied to the system deform it very differently depending on the size of the system. More specifically, the smaller the building blocks, the easier it is for level 0 squares to rotate which results in the very large increase in the value of $\theta_{0}$. On the other hand, for larger systems, the number of hinges corresponding to level 0 significantly increase, which makes it stiffer and promotes the rotation of level 1 building blocks as opposed to the rotation of individual level 0 squares. Of course, for very large systems, for example, corresponding to $N_{x}\times N_{y}=6\times 6$, the overall stiffness of the structure increases even further which reduces the extent of motion of the system. Based on Figure 5b, it is possible to note that the variation in the size of the structure also affects the Poisson’s ratio exhibited by the system. Upon having a closer look at the provided results, it is possible to note that structures composed of a very large number of square units are the most auxetic. This stems from the fact that large systems have many hinges associated with level 0, which makes it difficult for individual squares to rotate. Hence, level 1 building blocks retain a square-like shape throughout the deformation process. On the other hand, in the case of smaller systems, it is much easier for level 0 squares to rotate due to a smaller number of hinges present within the system, which results in level 1 building blocks assuming a rectangle-like shape. This in turn increases the value of the Poisson’s ratio.

All of this is very important as, in this work, it is shown that the considered hierarchical metamaterial is capable of exhibiting a controllable behaviour where, depending on the stiffness constant related to its hinges, it is capable of deforming in a very different manner. More specifically, by fine-tuning the magnitude of the stiffness constant, it is possible to obtain the structure where either level 0 or level 1 deforms to a greater extent. This in turn is very significant as it implies that the same in terms of its initial geometry structure can behave very differently and exhibit different values of the Poisson’s ratio solely based on the way how the hinges are designed. Furthermore, it is also discussed that the extent of such control over the behaviour of the structure can be further enhanced by introducing hybrid hinges to the system where hinges associated with the level 0 and level 1 correspond to a different stiffness constant. It is also important to emphasize the fact that the variation in the magnitude of the stiffness coefficient of hinges is not far-fetched and can be easily achieved in the case of the experimental prototype. For example, sides of neighbouring squares sharing a vertex can be connected to each other by means of an elastic material such as rubber. Then, depending on the amount of the rubber between the structural units, the stiffness of hinges could be easily controlled. This, in turn, can be achieved amongst others by means of standard SLA 3D printers. In addition, it is worth mentioning that the concept presented in this study can be extended to other hierarchical geometries, which can result in a greater range of mechanical properties of the already known materials.

The above discussion indicates that the results presented in this work make a valuable contribution to the state of art in the field of hierarchical mechanical metamaterials. This stems from the fact that, up to now, the dynamic behaviour of the specific hierarchical geometry analyzed in this work has been only studied by means of the highly idealised theoretical model [50]. Furthermore, the other studies [44,45,53] known in the literature were primarily focused on the quasi-static deformation of the system. This in turn does not allow for exploring all aspects of the possible deformation profiles and mechanical properties that can be observed throughout the deformation process. Thus, the possibility of the analysis of the time evolution of the considered hierarchical mechanical metamaterial by means of computer simulations certainly offers an interesting insight into what types of deformations processes it can undergo. In addition, in the case of the approach used in this work, we also consider a discrete mass distribution whose aspect was not taken into account in the other studies [44,45,53] related to the considered geometry. It is also important to emphasize the fact that, in this work, we investigated the possibility of using different types of hinges for specific hierarchical levels. In fact, this concept was already proposed in the literature, but, in the first paper where it was discussed [44], the extent of the mechanical deformation was very small, and it was not possible to analyze emergent deformation patterns. On the other hand, in the second study where this concept was analyzed [53], due to the constraints included in the model, the entire structure was deformed by external forces in a very different way than is the case in this work.

Finally, it is worth mentioning that the control over mechanical properties of the system considered in this work could prove to be useful in the case of numerous applications including protective devices where, depending on the cause of the mechanical deformation, one could use the material with a specific design of hinges in order to increase its efficiency. This stems from the fact that the appropriate design of hinges could enable the structure to exhibit a very specific variation in the profile of the Poisson’s ratio upon being subjected to mechanical deformation. Thus, depending on the type of the protective devices and the expected cause of the mechanical deformation, one could select a protective material where the design of hinges makes it possible for the structure to exhibit the most optimal deformation pattern. Hierarchical mechanical metamaterials similar to those studied in this work could also prove to be useful in the design of efficient biomedical devices such as stents, as suggested elsewhere [44]. In this case, the stent assuming and initially folded configuration upon being inserted into the blood vessel could expand differently depending on a specific patient.

## 4. Conclusions

In this work, it is shown that the behaviour and mechanical properties of the considered mechanical metamaterial can be conveniently controlled upon adjusting the magnitude of the stiffness coefficient corresponding to its hinges. More specifically, it is shown that either level 0 or level 1 of the structure can deform to a larger extent depending on the value of the stiffness constant. This in turn makes it possible for the same in terms of its initial geometry structure to deform in a variety of different ways. In this study, it is also shown that the control over the behaviour of the considered hierarchical system can be further enhanced by using different types of hinges to connect structural elements associated with level 0 and level 1 of the structure. It is also discussed that the deformation pattern and the magnitude of the Poisson’s ratio exhibited by the system can be controlled either via the stiffness coefficient associated with the hinges or a number of elements constituting level 1 building blocks. Thus, all of the presented results show a great potential of hierarchical mechanical metamaterials to be used in the design of versatile devices capable of adjusting their mechanical response depending on the particular application.

## Data Availability

The data are available from the corresponding author on reasonable request.
